# Reduced penetrance of Parkinson’s disease models

**DOI:** 10.1515/medgen-2022-2138

**Published:** 2022-08-12

**Authors:** Vanessa A. Morais, Melissa Vos

**Affiliations:** iMM, Instituto de Medicina Molecular, Faculdade de Medicina, Universidade de Lisboa, Lisboa, 1649-028, Portugal; Institute of Neurogenetics, University of Luebeck, Ratzeburger Allee 160 building 67, 23562 Luebeck, Germany

**Keywords:** Parkinson’s disease, reduced penetrance, animal models

## Abstract

The etiology and progression of Parkinson’s Disease (PD), the second most prevalent neurological disorder, have been widely investigated for several decades; however, a cure is still lacking. Despite the development of several neurotoxins and animal models to study this rather heterogeneous disease, a complete recapitulation of the neurophysiology and neuropathology of PD has not been fully achieved. One underlying cause for this could be that mutations in PD-associated genes have reduced penetrance. Therefore, the quest for novel PD models is required where a double hit approach needs to be evoked – a combination of genetic alterations and environmental factors need to be accounted for in one unique model simultaneously.

## Introduction

Parkinson’s Disease (PD) is the most common neurodegenerative movement disorder affecting approximately 2 % of the population over the age of 60 and is characterized by loss of nigrostriatal dopaminergic neurons and aggregation of alpha-synuclein enriched inclusions termed Lewy bodies [1]. The majority of PD cases are sporadic; however, approximately 5 % are related to genetic forms of this disorder. The identification of these causative genes and their PD-associated mutation has enabled the creation of animal models to aid in understanding the pathophysiology of PD. Studies using these animal models have dramatically increased our knowledge of PD; however, an animal model that represents all human characteristics has still yet to be developed. However, the discovery of familial forms of PD due to a single gene mutation [2] has led to the current paradigm of PD as a complex disease with both genetic and environmental contributions [3]. Age and a number of environmental exposure are known to be risk factors for the development of PD [4], [5]. Genetic variation is estimated to contribute approximately 25 % to the overall risk of developing PD [6], [7]. The genetic variants related to PD vary in terms of frequency and risk of PD. On the one hand, there are several rare variants in single genes that are pathogenic and sufficient to cause disease. These monogenic causes of PD were predominantly identified through linkage analysis of affected families. Examples of such genes include *SNCA*, *DJ-1* and *PRKN*. On the other hand, genome-wide association studies (GWAS) have identified large numbers of common genetic variants that individually contribute a small amount to the risk of developing PD.

The most common genetic cause of PD is mutations in *LRRK2*, of which the G2019S mutation is the most common. Interestingly, mutations in *LRRK2* are also found in patients suffering from sporadic forms of PD [8] and the age of onset of LRRK2-dependent PD is affected by environmental and lifestyle factors [9], suggesting that other factors play a role in the development of the disease and thus support the existence of reduced penetrance in PD. Furthermore, and adding an extra level of complexity to understanding the molecular mechanism that underlies PD is the fact that the genetic landscape of PD is characterised by rare high penetrance pathogenic variants causing familial disease (such as SNCA, PINK1, DJ-1) and high frequency, low penetrance variants (such as GBA). Variants with high penetrance are very rare, whereas those with variable penetrance (such as LRRK2, GBA) are common worldwide, particularly in specific populations.

Hence, key factors in the underlying mechanisms of this disease remain elusive; therefore, current treatment mainly focuses on alleviating PD-related symptoms. Interestingly, the proportion of carriers of disease-causing mutation that do not develop the disease is surprisingly large [10], which adds to the lack of a complete understanding of the disease pathophysiology.

## Modeling of PD

As PD is a rather heterogeneous disease with a varying age of onset, symptoms and rate of progression and moreover, as brain pathology in humans can only be confirmed by examining post-mortem tissue [11], there is a great need for experimental animal models to deepen our understanding of this multifaceted disease.

### Species-specific characteristics

Like most human diseases, the three animals preferentially used to model PD are rodents, non-human primates and non-mammalian species. Each animal has species-specific advantages and limitations that need to be taken into account when assessing behavioural and pathophysiological findings. Rodents are by far the most extensively used animal model across all biomedical fields as they are convenient to house and care for in laboratory conditions and robust experimental protocols that include different forms of drug administration, generation of transgenic strains and behavioural assessments. An advantage of the PD rodent models is that the dopaminergic degeneration correlates with observed motor deficits in rats and mice [12]. These motor deficits are attained by performing a battery of behavioural tests, most of which involve measuring movement, grip, and strength of front paws [13], [14], [15]. Studies performed in non-human primates give invaluable insight into PD pathology due to their anatomic and genetic similarity to humans. However, due to their longer life span, demanding care and maintenance costs, and complex ethical considerations, studies in these animal models are very often reserved for preclinical evaluation of therapies [16]. Non-mammalian models, such as *Drosophila melanogaster* and *C. elegans*, have been used in the past decade in several PD studies. These models present several advantages, such as fast genetic manipulation, a rapid reproductive cycle, low maintenance costs, and most importantly, a well-defined neuropathology and behavioural pattern [17], [18]. For example, *C. elegans* models have a fully mapped connectome possessing only 302 neurons, out of which only eight are dopaminergic, while *Drosophila* has a larger connectome containing 135,000 neurons but is still being mapped.

### Neurotoxin models

For PD, in specific, two main approaches are used to model this disorder in experimental models: a neurotoxin approach, where the loss of dopaminergic neurons arising from environmental factors can be modelled; or a genetic approach, where cell loss and motor defects can be assessed.

For the neurotoxin models, dopaminergic neuron degeneration is induced by local or systemic administration of neurotoxins. The 6-hydroxydopamine (6-OHDA), a dopamine analogue, is a respiratory chain Complex I inhibitor that was one of the first PD-associated neurotoxins to be discovered [19] and has been widely used to model PD in rodents and non-human primates [20], [21]. Another PD-associated neurotoxin, 1-methyl-4-phenyl-1,2,3,6-tetrahydropyridine (MPTP), was discovered through a failed opioid drug synthesis process where the consumer group developed parkinsonism clinical features [19], [20]. MPTP, also a Complex I inhibitor, has been used to model PD in mostly rat and mouse models in a dose-dependent manner [22] and is considered to be the gold standard for PD models in non-human primates [21]. Additionally, one of the most debated neurotoxins used to model PD is pesticides and herbicides [23], [24]. Emphasis has been made on the pesticides rotenone and paraquat, both inhibitors of the mitochondrial respiratory chain; however, a conclusive correlation between agrochemical exposure in populations and increased risk for PD has still to be clarified [25]. Nevertheless, pesticide models can increase the understanding of how environmental factors can affect PD risk.

### Genetic models

Genetics plays an important role in PD pathogenesis. Disease-causing mutations have been identified through familial PD linkage analysis, while genetic risk factors for idiopathic PD have been identified through association analysis between patients and controls [26]. Autosomal dominant and autosomal recessive mutations, both with variable penetrance, have been associated with familial PD. However, de novo mutations in the PD-linked genes have also been identified in patients lacking a PD family history. *SNCA* was the first gene linked to familial PD and its encoded protein alpha-synuclein is one of the main constituents of Lewy bodies [2], [27]. At present, three autosomal dominant *SNCA* point mutations that are fully penetrant have been identified: A53T, A30P and E46K [28], [29]. Concerning autosomal dominant mutations, *LRRK2* was identified in 2004 as a monogenic cause of PD [30], being the G2019S and the R1441C/G mutation the most common ones. However, the *LRRK2* mutations display an incomplete and varying penetrance depending on the population of origin, meaning that not all mutation carriers will develop PD [31]. *UCH-L1* mutations have also been linked to autosomal dominant forms of PD [20].

Autosomal recessive mutations in *Parkin*, *PINK1* and *DJ-1* have been associated with familial PD. *Parkin* is the most commonly mutated gene in early-onset PD, accounting for approx. 50 % of familial cases and 20 % of idiopathic cases of PD [32]. *PINK1* is the second most common mutated gene linked to early-onset PD, representing approx. 1–7 % of the cases. *DJ-1* mutations are more uncommon [33]. Interestingly, rare individuals that have mutations in two of these three genes appear to have a complete penetrance [28], [32], [34].

In sum, although many efforts have been made to replicate familial PD by generating transgenic mice carrying these autosomal dominant or recessive mutations, these models do not display a clear dopaminergic neurodegeneration or parkinsonian motor deficits [28], [35], thus, do not mimic the neuropathology of PD. Therefore, not only a quest for improved models is an essential requirement for this field, but species-specificity and mutation penetrance should also be taken into consideration when developing novel research tools to study the neuropathology and physiology of this disorder.

## Factors affecting penetrance

**Figure 1:**
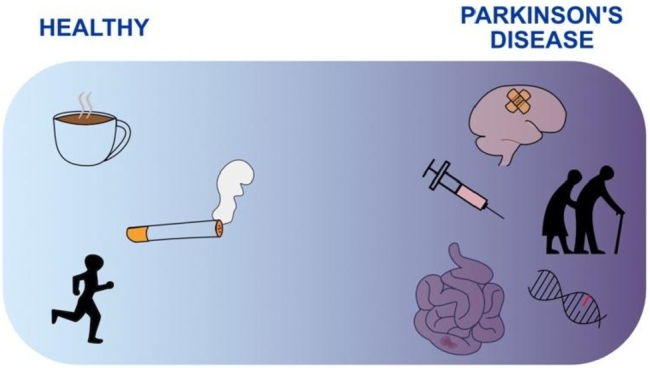
Signs and symptoms of Parkinson’s disease vary in severity. Several factors have been identified that affect the penetrance of PD. Caffeine, smoking cigarettes and physical activity have a beneficial effect, while brain injury, drugs, age, inflammation and gene mutations (e. g. GBA) enhance or worsen PD.

An interesting observation, as mentioned above, is the lack of dopaminergic neuron loss in rodent (and partially *Drosophila*) models and strong phenotypes in rodent PD models [28], [35]. A possible explanation can be found in the presence of factors that affect the penetration of PD signs or the presence of a multiple hit model for PD in which multiple risk factors combined converge to PD. We will discuss the most common elements that alter the penetrance of PD and the use of animal models to study the underlying mechanisms (Figure 1).

### Risk factors for PD

PD incidence increases with age and thus, aging constitutes an important risk factor. With a population that is increasingly becoming older, PD exhibits pandemic characteristics [1]. In addition, age of onset is a highly variable factor, suggesting that other factors play an important role, including lifestyle and environmental factors [36]. Nonetheless, aging in the fruit fly results in neurodegeneration that is, however, not specifically to dopaminergic neurons, further suggesting that additional factors are important to induce PD. Furthermore, the fruit fly is a relatively short-lived animal and hence, other animal models such as rodent and non-human primate models provide a better model to study the effect of aging on the cellular mechanisms that result in dopaminergic neurons.

The most common genetic risk factor for PD is Glucocerebrosidase (GCase) [37], [38], encoding the lysosomal enzyme GCase. Homozygous loss of *GCase* results in the lysosomal storage disorder Gaucher’s disease, while the heterozygous mutations increase the risk for the development of PD [39]. More than 100 mutations have been identified in *GCase* in which L4440 and N370S are the most commonly found in PD, contributing to more than 70 % in a north-African population [38], [40]. Studies in animal models found that co-expression of heterozygous mutant *GCase* and mutant *SNCA* exacerbated the observed symptoms compared to the expression of alpha-synuclein alone [41], further providing evidence that mutant *GCase* increases the risk for PD. However, the underlying mechanisms remain unclear as some report a gain of function of GCase resulting in PD, while others describe the loss of GCase as the underlying mechanisms [42]; thus, further studies are required to understand how heterozygous loss of GCase results in increased risk for PD. In addition, different mutations in *GCase* are linked to a higher or lower risk for PD [38], [43], suggesting that the nature of the *GCase* mutations is important and studies in animal models focusing on the various effects of the different mutations would strongly add to our understanding of the underlying mechanisms.

Many studies report a correlation between PD and environmental factors such as pesticides and insecticides [44], [45]; however, a lack of consistency was identified following a systematic review analysis [46]. These studies are sometimes difficult to interpret as exposure to environmental factors potentially occurs years prior to the initiation of symptoms and, therefore, might be disregarded. Nonetheless, and as mentioned above, supplementation of pesticides in the medium of animal models has further contributed to the elucidation of underlying mechanisms in PD, more specifically to the mitochondrial complex I defect [25].

Mild traumatic brain injury is correlated with an increased risk for PD [47], [48]. Interestingly, a common feature between PD and brain injury is elevated pro-inflammatory markers [49], [50], implying that increased inflammation following brain trauma adds to an increased risk of developing PD. Remarkably, LRRK2 was found to be upregulated in mice traumatic brains and inhibition of LRRK2 appears to be neuroprotective [51].

### Inflammation as part of a second hit model in PD

Lewy bodies, the pathological hallmark of PD, are surrounded by immunoglobulins suggesting an active inflammatory response [52]. Furthermore, recently the importance of inflammation in PD has emerged and is supportive of a multiple hit model to be present in PD pathogenesis. Furthermore, pro-inflammatory markers are elevated in the serum of patients suffering from PD [50]. Several studies report a function of LRRK2 in neuroinflammation and LRRK2 mutations enhance pro-inflammatory response [53]. Interestingly, the G2019S LRRK2 mutation is abundantly present in both sporadic and familial forms of PD [54]; however, it has a surprisingly low penetrance [53], further supporting the existence of a second hit model for PD that involves inflammation. Interestingly, other genes involved in PD, including PINK1 and Parkin, function in immunity [55], further supporting the important role of inflammation in PD.

In addition, following exhaustive exercise, these mice knockout for Parkin or Pink1 show increased inflammatory response, an observation that was also found in serum from patients with PD [50]. The observed inflammatory response can be prevented by blocking STING, a central regulator of the type I interferon response [50]. Furthermore, loss of STING rescued the dopaminergic neuron loss and motor defects in aged Parkin knockout mutator mice that accumulate mutations in mitochondrial DNA [50]. However, it remains unclear to what extent an inflammatory response is sufficient to induce dopaminergic neuron loss and motor impairment in Parkin or Pink1 knockout mice. Interestingly, loss of *Drosophila* Sting and its downstream effector Relish in Pink1- or Parkin-deficient flies does not improve the behavioural and mitochondrial abnormalities in these flies [56], further puzzling the role of stimulated inflammatory activation in the manifesting disease phenotypes. A possible mechanistic explanation comes from a study in Pink1 knockout mice that were infected with bacteria in the intestines resulting in an autoimmune response. The provoked cytotoxic T cells are also present in the brain and induced motor impairment that was rescued by L-DOPA treatment. Furthermore, these T cells exhibited the capacity to kill dopaminergic neurons [57]. These findings support the role of PINK1 as an immunosuppressant and highlight the importance of the brain-gut axis in PD, which was further supported by the recent identification that the probiotic PXN21 strain of the gut bacteria Bacillus subtilis inhibits alpha-synuclein aggregation in *C. elegans* [58].

### Beneficial factors: smoking, coffee, physical activity

In addition to risk factors, protective elements have been identified that delay the onset of PD or result in less severe symptoms. The most surprising is that cigarette smoking which is the cause of numerous cancers, appears to be protective against PD [46]. The question arose if this was due to the nicotine or other factors that were involved in cigarette smoking. Experiments in PD fly models showed that nicotine-free tobacco exerts a neuroprotective effect by modulation of the nuclear factor erythroid 2- related factor 2, NRF2 [59]. NRF2 functions in many processes, including mitochondrial physiology and immune responses [60], two processes also involved in PD. Interestingly, the expression of NRF2 was increased in mitochondrial fibroblasts derived from PD patients carrying the LRRK2 G2019S mutation [61]. While these data suggest nicotine does not contribute to the protective role of smoking in PD, other studies in various PD models show a protective role of nicotine functioning via the cytochrome P450 enzymes [62]. These studies show higher levels of the cytochrome P450 2D6 that affect drug metabolism and lead to the inactivation of neurotoxins [63]. Secondly, lifelong caffeine use has been linked to a lower risk and a delayed onset of PD and caffeine application is neuroprotective in several PD models [36], [64]. Caffeine is an antagonist of adenosine receptors [65] and confers its neuroprotection by altering neuroinflammation, mitochondrial function and autophagy [64]. In flies, caffeine was also shown to be involved in wake-promoting in a dopamine-dependent fashion [66], providing an alternative or additional explanation for the beneficial effect of caffeine. Finally, physical exercise is in general beneficial for people’s health and has been shown to alleviate PD symptoms and serves as an addition to pharmacological therapy [67]. Interestingly, in toxic PD animal models, physical exercise revealed to be neuroprotective, reduced neuroinflammation and oxidative stress [68], suggesting that physical exercise is not only a factor that alleviates symptoms during the disease process but also has the capacity to delay or reduce the severity of symptoms in PD, although the exact underlying mechanism remains elusive. The application of exercise studies in *drosophila* is relatively novel [69] and can provide a new tool to increase our knowledge of the beneficial mechanisms of physical exercise.

## Future perspectives

Studies using animal models have already greatly added to our knowledge of the underlying mechanisms in PD; however, many questions still remain that can be viewed in the light of reduced penetrance. Hence, to understand the effect of reduced penetrance, it is important to address this when studying PD in animal models. Several approaches are possible that will be addressed below.

### Double hit approach

It is clear that risk or protective factors play an important role and exert a significant impact on the disease progression. Hence, it is key to understand the underlying mechanisms that alter the penetrance of the disease symptoms. Research in animal models has already significantly contributed to our knowledge; however, important questions regarding the disease pathogenesis remain unanswered and therefore, a therapy to stop or reverse the disease has not yet been developed. An increasing amount of evidence supports the role of PD being a double hit model and thus, an all-inclusive approach is highly recommended. An important example of this is the combination of loss of Parkin and the induction of mitochondrial mutations via a mutator mouse that resembles the effects of aging on mitochondrial DNA. Thus, this double hit model combines aging and genetics and provokes dopaminergic neuron loss, its accompanying motor deficits and pro-inflammatory response. Interestingly, a defective circadian rhythm is often concomitant with the development of PD and studies claim it to be an early, unrecognized symptom of the disease. Loss of Pink1 or Parkin in flies displays similar defects in the circadian rhythm [70]. Furthermore, studies in flies linked caffeine to the dopaminergic system [71], providing a possible explanation of how these mechanisms are linked.

Thus, two factors appear commonly in elements affecting reduced penetrance, nl., inflammation and mitochondrial function, further underlining the importance of these mechanisms. Hence, in our attempt to further understand the underlying mechanisms of PD, an approach that includes a combination of different factors is key.

### Develop a screen approach

*Drosophila* and *C. elegans* are the animal models of choice for performing genetic screens to identify alleles that will increase or reduce the penetrance of PD-related symptoms. Both models are prone to genetic modification and the application of the yeast UAS-Gal4 system allows up or downregulation for gene-wide screens in a feasible fashion [72]. Hence, screens with mutations in a PD-related background can provide the identification of novel genes or pathways to be involved in altered penetrance in PD. Furthermore, the effects of gene dosage can be tested by overexpression or knockdown of a specific gene. In this regard, loss of Heixuadian was identified in a genetic modifier screen to be enhancing the observed phenotypes in *pink1*-mutant flies. However, overexpression of Heixuedian is involved in the synthesis of the lipid-soluble vitamin K2 in Pink1-deficient flies resulting in a rescue of the phenotypes [73]. Thus, gene dosage variations can have a strong impact on the phenotypes.

Even though genetic screens are a useful tool, the disadvantage is that it does not allow the finetuning of protein expression levels. For this, dose-response screens can be applied. However, the application of these screens in animal models will only provide information for the specific animal model and translatability to human patients can be challenging. Drug screens, similarly to genetic screens, hold strong potential to identify novel protective or enhancing benefits. Furthermore, drug screens can further build upon data attained from the genetic screens, which is already a step closer to being on the correct path to achieving a feasible drug.

### Benefit from NGS for the identification of novel players

Whole exome sequencing is increasingly used for diagnostic purposes; however, also for the understanding of the disease mechanisms, it proves its value. Genetic screens have greatly benefited from next-generation sequencing as it allows the identification of specific mutations or altered RNA expression resulting in altered penetrance. Furthermore, the progressive advances in NGS have allowed genome-wide association studies in which alterations in big cohorts can be identified and thus, with this also pathways that are affected in PD. This way, several pathways that are affected in PD have been identified, likewise the lysosomal pathway [74]. In addition, systematic meta-analyses allow the identification of risk loci [75], including the sterol regulatory element-binding transcription factor 1 (SREBF1). A genome-wide RNAi screen in *Drosophila* confirmed SREBF1 to be a risk factor via its regulatory function in mitophagy [76].

## Conclusions

Studies using animal models to investigate the underlying mechanisms in PD have paved the road for the presence of reduced penetrance or a second hit model to induce PD; however, only recently have the efforts been increased to further elaborate on this. Animal models can be used to tackle several points upon reduced penetrance or the existence of a second hit: 1. the identification of additional factors in PD; 2. the study of the underlying mechanisms by using a second hit which will enable an increased knowledge on the pathogenesis of PD; and 3. drugs can be tested in animal models for their efficacy in postponing or preventing the onset of PD.
